# Total Hip Arthroplasty after Treatment of Pseudojoint Infection in a Patient with a Highly Dislocated Hip

**DOI:** 10.1155/2013/947121

**Published:** 2013-06-25

**Authors:** Kyung-Soon Park, Jong-Keun Seon, Seon-Yoon Nah, Taek-Rim Yoon

**Affiliations:** Department of Orthopaedic Surgery, Chonnam National University Hwasun Hospital, Chonnam National University Medical School, 322 Seoyong-ro, Hwasun-eup, Hwasun-gun, Jeonnam, Chonnam 519-809, Republic of Korea

## Abstract

Infection at the pseudoacetabulum in a patient with a high hip dislocation has not been reported previously in the English literature. We report a case of total hip arthroplasty in a 28-year-old female who presented to us with hip pain following debridement of the infected pseudojoint in a case of neglected developmental dysplasia of the hip. The infection was treated with thorough debridement and drainage. However, even after achieving complete infection control, this patient complained of disabling right hip joint pain. Total hip arthroplasty with subtrochanteric osteotomy was performed to relieve the pain and improve gait. After surgery, the patient's symptoms were relieved. We consider that in this case of acute pseudojoint infection simple arthrotomy and debridement combined with irrigation and drainage provide effective treatment. But muscle weakness and more increased joint laxity can cause hip pain even after infection control. So total hip arthroplasty is likely to be necessary after the infection has been controlled in a patient with a highly dislocated hip.

## 1. Introduction

High dislocation of adult hip as sequelae of developmental dysplasia of the hip (DDH) presents with a distorted femoral head located in the pseudoacetabulum and leg length discrepancy [1–4]. Furthermore, contracture and shortening of surrounding neurovascular structures increase the likelihood of injury during surgery [3]. These problems have been frequently discussed, but infection at the pseudoacetabulum in high hip dislocation and its treatment have not been previously reported in the English literature. Here, we report a patient with an acute infection at the pseudoacetabulum with high hip dislocation. After the treatment of the infection, this patient was underwent total hip arthroplasty (THA). The patient agreed that her case data could be submitted for publication.

## 2. Case Report

**Figure 1:**
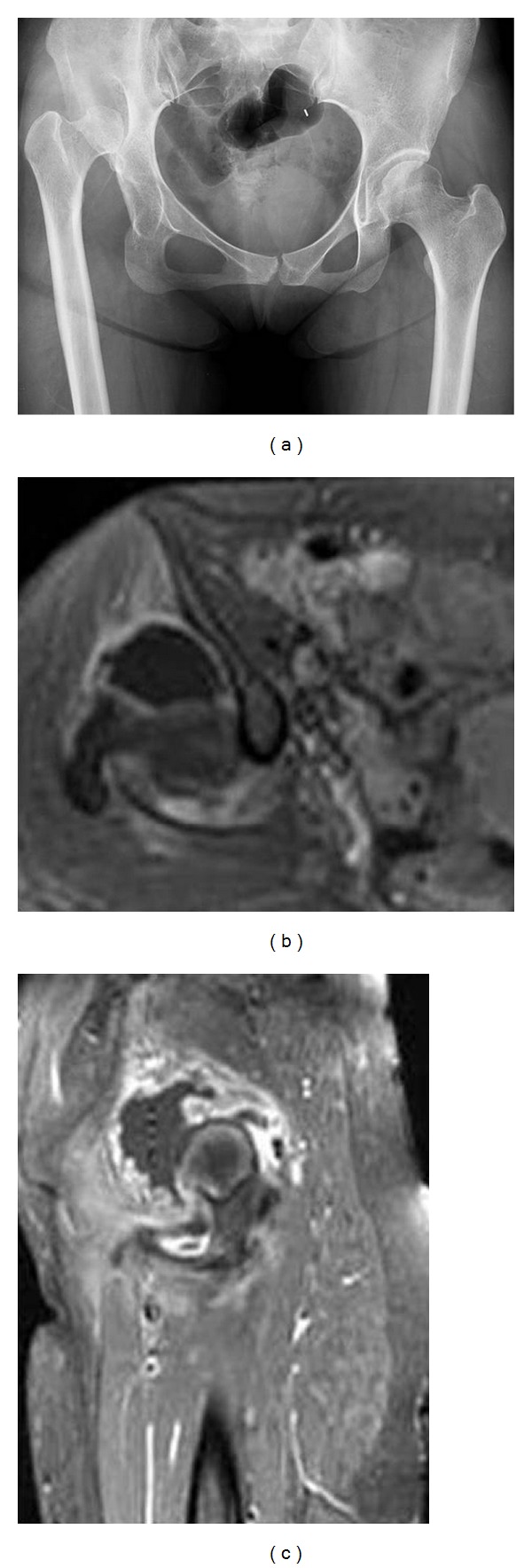
(a) Preoperative hip anteroposterior radiograph of a 28-year-old woman shows right hip high dislocation, a small femoral canal, and a pseudoacetabular joint. (b) and (c) Preoperative axial and sagittal T2 weighted enhanced MR images of the right hip joint show an inflammatory reaction with huge joint effusion and synovial thickening.

A 28-year-old woman was transferred to our emergency room for severe right hip pain and a febrile sensation of 5-day duration. A physical examination of the right hip joint revealed swelling, tenderness, and painful range of motion limitation. Radiography showed right hip high dislocation and dysplasia with small femoral canal and head and pseudoacetabular joint (Figure 1(a)). MRI showed inflammatory reaction with joint effusion and synovial thickening with rim enhancement (Figures 1(b) and 1(c)). Her laboratory findings revealed white blood cells (WBCs) 8700/mm^3^ (neutrophils 83.8%), erythrocyte sedimentation rate (ESR) 93 mm/hr, and C-reactive protein (CRP) 13.45 mg/dL, suggesting the presence of acute infection. Emergency surgery was undertaken and thorough debridement and irrigation with drain insertion were performed through a posterolateral approach.

Gram staining and culture of a pus discharge encountered intraoperatively revealed the presence of Gram-positive cocci and *S. aureus*, respectively. From the 1st postoperative day 1 gram of cefazolin was administered t.i.d. for 3 weeks followed by oral antibiotics (cefroxadine 250 mg) t.i.d. for 2 weeks. CRP normalized at 3 weeks postop and ESR 2 weeks later. Drainage was removed when the daily amount of fluid was less than 10 cc (2 weeks postop). She was discharged from the hospital at 3 weeks postop and she was followed for 7 months. However, at 7 months after surgery, she still complained of right hip pain and severe functional impairment, but there were no signs of infection and her laboratory findings were normal. Initially, she was treated conservatively for the pain with NSAIDs and rest but the pain continued. Accordingly, we decided on THA at about 10 months after initial surgery. 

**Figure 2:**
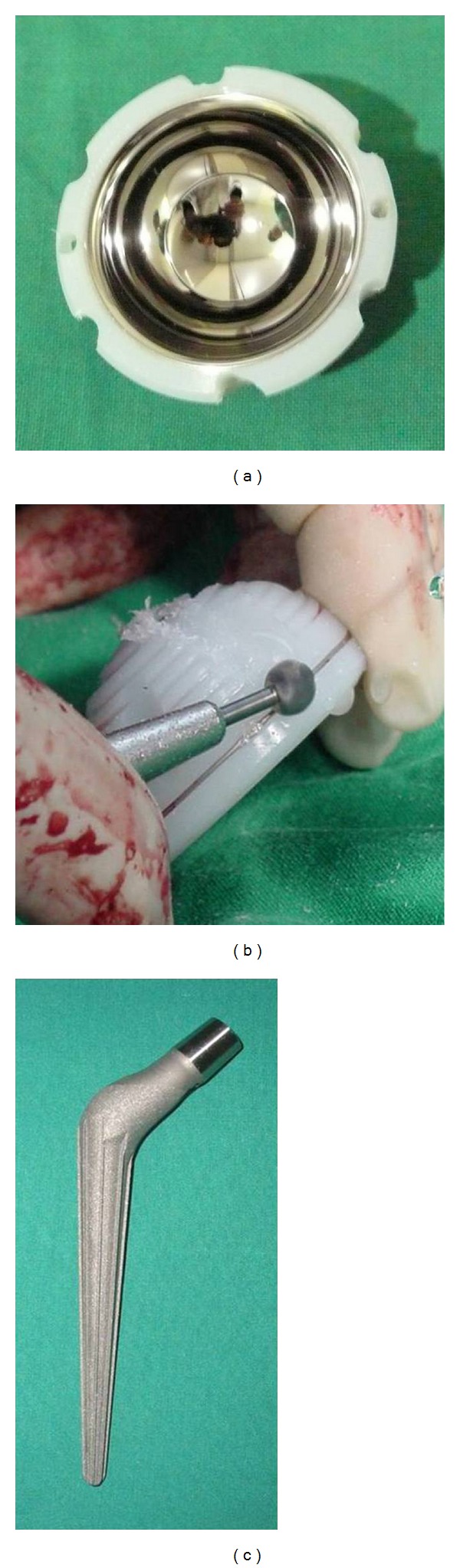
(a) and (b) The back surface of metal inlay polyethylene liner was downsized and roughened in a spider web manner with an electrical burr. (c) Fully coated, porous cone prosthesis with 8 ribs for rotational stability.

THA was performed through a posterolateral approach in the lateral position and the absence of infection was confirmed by frozen biopsy. A provisional transverse femoral osteotomy was performed at the level of lesser trochanter and the operation field was adequately exposed. The true acetabulum was too small to insert a normally sized acetabular cup, and thus, we undersized the outer diameter of a metal inlay polyethylene (Metasul, Centerpulse Orthopedics, Austin, TX, USA) liner using an electrical burr to obtain adequate liner containment in the small acetabulum (Figures 2(a) and 2(b)). Before inserting the liner with cementing into the acetabulum, medial wall osteotomy was performed and autologous chip-bone graft was done. 

Because the femoral canal was dysplastic, we used a small diameter Cone Prosthesis (Protek AG, Berne, Switzerland) (Figure 2(c)). After final reduction, the greater trochanter was reattached to the proximal femur with cables (Dall Miles, Stryker Orthopaedics Inc., Mahwah, NJ, USA). 

**Figure 3:**
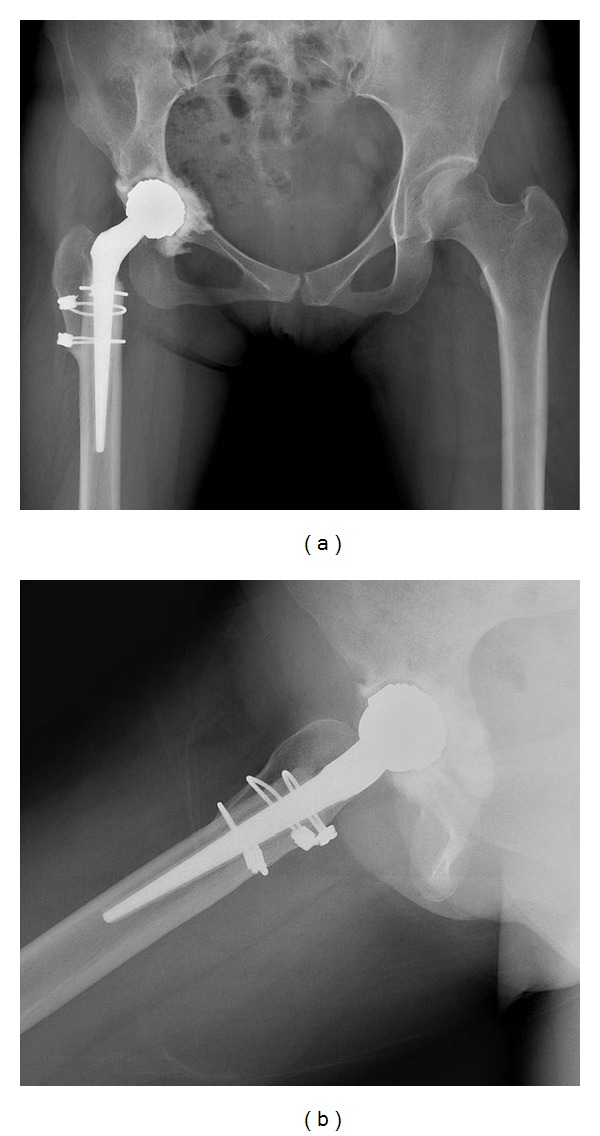
Postoperative radiographs taken 5 years 3 months after THA. (a) Both hip anteroposterior and (b) right hip lateral radiographs show complete bone union at the osteotomy site and no evidence of osteolysis.

Range of motion exercise and nonweight bearing two-crutch walking were started from 2nd postoperative day. At 3 months, she started to walk without any support and abductor-strengthening exercises were started. Now she followed up 5 years 3 months postoperatively and she had a complete and painless walk and range of motion. But there was about 1.5 cm of leg shortening (Figures 3(a) and 3(b)).

## 3. Discussion

Primary septic arthritis of the hip joint is usually encountered in children but may occur in adults, particularly in those with a debilitating disease or sepsis at another site [5]. Untreated septic arthritis results in bone destruction, avascular necrosis, growth abnormalities, and damage to articular cartilage [6–8], and thus, the treatment goal is always to attempt limb salvage and preserve joint function. The treatment of septic arthritis in adult hip joints requires early arthrotomy with thorough debridement and irrigation. Antibiotics are the mainstay treatment of acute septic arthritis, but chronic septic arthritis is invariably complicated by osteomyelitis and cartilage destruction, and thus, a staged approach with initial clearance followed by reconstruction is required [9]. 

On presentation, our patient had both local and systemic symptoms of acute infection, that is, pain, warmth, fever, and an increased CRP level with a symptom duration of 5 days. Accordingly, she was diagnosed as having an acute hip joint infection. Because the infection was confined in the pseudojoint by MRI and cartilage status of femoral head appeared not to be important given her hip joint function, we decided on arthrotomy with debridement and irrigation. Furthermore, because the joint capsule was lax due to hip joint dysplasia and dislocation as evident by MRI, which showed a huge amount of joint effusion, we considered that the joint pressure was not high enough to compromise blood supply to the femoral head. 

Emergent surgery and antibiotics were effective and her laboratory findings normalized at 2 weeks, but even after eradication of the infection, at 7 months postop she complained of more disabled right hip and pain. 

During the follow-up period after arthrotomy, she continued with abductor and quadriceps muscle strengthening exercises, but these were ineffective, and thus, we decided on THA. We believe now that the disabled right hip and pain were due to weakened muscles and increased joint laxity due to previous joint capsulotomy and debridement. 

Contracture and shortening of surrounding neurovascular structures increase the likelihood of injury during surgery on highly dislocated hips, and the restoration of an anatomic hip center is one of the goals of THA in such patients. However, this process may require leg lengthening, which increases the risk of neurovascular injury to as much as 13% [3]. Subtrochanteric femoral shortening osteotomy is now widely used for THA in high-dislocated hips. We used Paavilainen's osteotomy technique and a small conical stem due to the straight, relatively narrow nature of the dysplastic femur, and because this technique is easier, it provides earlier bony union at osteotomy sites and achieves similar levels of leg lengthening [10]. Furthermore, we used a fully coated, porous cone prosthesis with 8 ribs for rotational stability and were able to restore femoral anteversion easily. Later, we observed bone ingrowth of the greater trochanter into the conical stem. 

In terms of acetabular reconstruction, it is important that the normal center of hip rotation be reestablished, as the nonanatomical placement of a component is an important predictor of acetabular loosening. In our patient, because the acetabular bone stock was too deficient to insert a metallic acetabular cup, we performed cotyloplasty and medial bone grafting and used cement to fix the metal inlayed liner.

In this patient with high hip dislocation due to DDH, we successfully treated an acute pseudojoint infection and performed THA with subtrochanteric osteotomy. We consider that, in cases of acute pseudojoint infection, simple arthrotomy and debridement combined with irrigation and drainage provide effective treatment and that THA is likely to be necessary after the infection has been controlled.
